# A high‐throughput BAC end analysis protocol (BAC‐anchor) for profiling genome assembly and physical mapping

**DOI:** 10.1111/pbi.13203

**Published:** 2019-07-15

**Authors:** Xiaohui Yang, Yu Yang, Jian Ling, Jiantao Guan, Xiao Guo, Daofeng Dong, Liping Jin, Sanwen Huang, Jun Liu, Guangcun Li

**Affiliations:** ^1^ Vegetable and Flower Research Institute of Shandong Academy of Agricultural Sciences Molecular Biology Key Laboratory of Shandong Facility Vegetable National Vegetable Improvement Center Shandong Sub‐Center Huang‐Huai‐Hai Region Scientific Observation and Experimental Station of Vegetables Ministry of Agriculture and Rural Affairs Jinan China; ^2^ Institute of Vegetables and Flowers Chinese Academy of Agricultural Sciences, Key Laboratory of Biology and Genetic Improvement of Tuber and Root Crop Ministry of Agriculture and Rural Affairs Beijing China; ^3^ Institute of Crop Science Chinese Academy of Agricultural Sciences National Key Facility for Crop Resources and Genetic Improvement Beijing China

**Keywords:** BAC end library, BAC‐anchor, whole‐genome profiling, heterozygosity, autotetraploid potato

## Abstract

Traditional approaches for sequencing insertion ends of bacterial artificial chromosome (BAC) libraries are laborious and expensive, which are currently some of the bottlenecks limiting a better understanding of the genomic features of auto‐ or allopolyploid species. Here, we developed a highly efficient and low‐cost BAC end analysis protocol, named BAC‐anchor, to identify paired‐end reads containing large internal gaps. Our approach mainly focused on the identification of high‐throughput sequencing reads carrying restriction enzyme cutting sites and searching for large internal gaps based on the mapping locations of both ends of the reads. We sequenced and analysed eight libraries containing over 3 200 000 BAC end clones derived from the BAC library of the tetraploid potato cultivar C88 digested with two restriction enzymes, *Cla* I and *Mlu* I. About 25% of the BAC end reads carrying cutting sites generated a 60–100 kb internal gap in the potato DM reference genome, which was consistent with the mapping results of Sanger sequencing of the BAC end clones and indicated large differences between autotetraploid and haploid genotypes in potato. A total of 5341 *Cla* I‐ and 165 *Mlu* I‐derived unique reads were distributed on different chromosomes of the DM reference genome and could be used to establish a physical map of target regions and assemble the C88 genome. The reads that matched different chromosomes are especially significant for the further assembly of complex polyploid genomes. Our study provides an example of analysing high‐coverage BAC end libraries with low sequencing cost and is a resource for further genome sequencing studies.

## Introduction

Polyploidization has played a predominant role in plant diversification and speciation (Feldman *et al*., 2012; Tang *et al*., 2008). Around 40% of angiosperms and 54% of crops are polyploid species, and approximately 70% underwent polyploidization during their evolution (Feldman *et al*., 2012; Masterson, 1994; Salman‐Minkov *et al*., 2016). Genomic studies have revealed that polyploidy also increases allelic diversity and heterozygosity, as well as enhances meiotic recombination, which can facilitate the selection and improvement of adaptive traits under cultivation conditions (Ross‐Ibarra *et al*., 2007; Salman‐Minkov *et al*., 2016). In the evolutionary process of polyploidization, many plants have developed large and complex genomes, especially domesticated crops. Although it is still a technical challenge to assemble the chromosome sequences of these polyploid species, a feasible and widely used genome sequencing strategy is to assemble the genome of their diploid relatives and/or their doubled‐haploid lines, the so‐called ‘haploid model genotypes’. Efforts over the past decade have led to the assembly of the genome sequences of haploid genotypes derived from several important polyploid plants, such as triploid banana (D'Hont *et al*., 2012), tetraploid potato (PGSC, 2011), tetraploid tobacco (Sierro *et al*., 2014), tetraploid cotton (Li *et al*., 2014) and hexaploid wheat (Ling *et al*., 2013; IWGSC, 2014). The availability of these haploid reference genomes has greatly accelerated genomic and genetic studies in polyploid plants.

Potato (*Solanum tuberosum* L.) is the third most important food crop in the world after wheat and rice, with an annual global production exceeding 374 million tons (CIP, 2016). Tubers are primary storage organs and function in vegetative reproduction due to their high content of starch, protein, minerals, vitamins and antioxidants. Cultivated potato is an autotetraploid (2n = 4x = 48), with high heterozygosity and acute inbreeding depression; these attributes prevent the use of classical breeding approaches to improve potato. Based on the genome sequence of the haploid potato (PGSC, 2011), Chinese scientists have been committed to the cultivated potato genome sequencing project using Cooperation‐88 (C88), a high‐yielding autotetraploid potato cultivar that is resistant to *Phytophthora infestans* and also has excellent virus resistance (Li *et al*., 2011). Homologous assembly and joining are often challenging due to the complex architecture and high content of repetitive sequences found in this potato cultivar. In a previous study, we constructed a BAC library that contained more than 800 000 clones with an average insert size of 90 kb based on CopyControl^™^ pCC1 vector (Epicentre) (Yang *et al*., 2015). This BAC library has been verified for quality and used for genome sequencing and assembly of the tetraploid potato.

In order to do BAC library‐based genome assembling, physical map construction, BAC end marker development and so on, it is necessary to sequence BAC ends of the whole library. This process is often laborious and expensive using traditional sequencing approaches, which limits a better understanding of the genomic features of polyploid plants, including potato. There have been two paired‐end profiling strategies for large‐insert genomic libraries: Fosill for Fosmid libraries and pBACode for BAC libraries. However, Fosill is less suitable for long genomic end sequences because the co‐ligation of the two ends is much more difficult, which impedes self‐circularization. Fosill is more suitable for short‐end sequences, but the short ends generated with Fosill cannot be used with long‐read high‐throughput techniques (Williams *et al*., 2012). pBACode utilizes PCR to generate 1:*N* paired barcodes, which are comprised of multiple vectors with the same barcode on one end, and larger BAC libraries have a higher fraction of 1:*N* paired barcodes (Wei *et al*., 2017).

Here, we developed a highly efficient, less expensive BAC end identification protocol, BAC‐anchor analysis, using the Illumina^®^ high‐throughput sequencing platform. BAC‐anchor was mainly developed for measuring BAC library quantity, BAC end pairing analysis and genomic survey analysis in polyploid species; these measurements are required for further genome assembly, scaffold‐joining and gap‐filling analysis. Our approach focused on the identification of high‐throughput sequencing reads harbouring restriction enzyme cutting sites. In brief, we used the gapped mapping protocol to align paired‐end reads derived from BAC end libraries to draft chromosome sequences, scaffolds and/or the genome sequences, which enabled us to search for ultra‐long paired‐end reads containing large internal gaps. Our study provides a potato BAC resource and a highly efficient protocol for further genome assembly studies.

## Results

### BAC‐anchor and BAC end libraries

**Figure 1:**
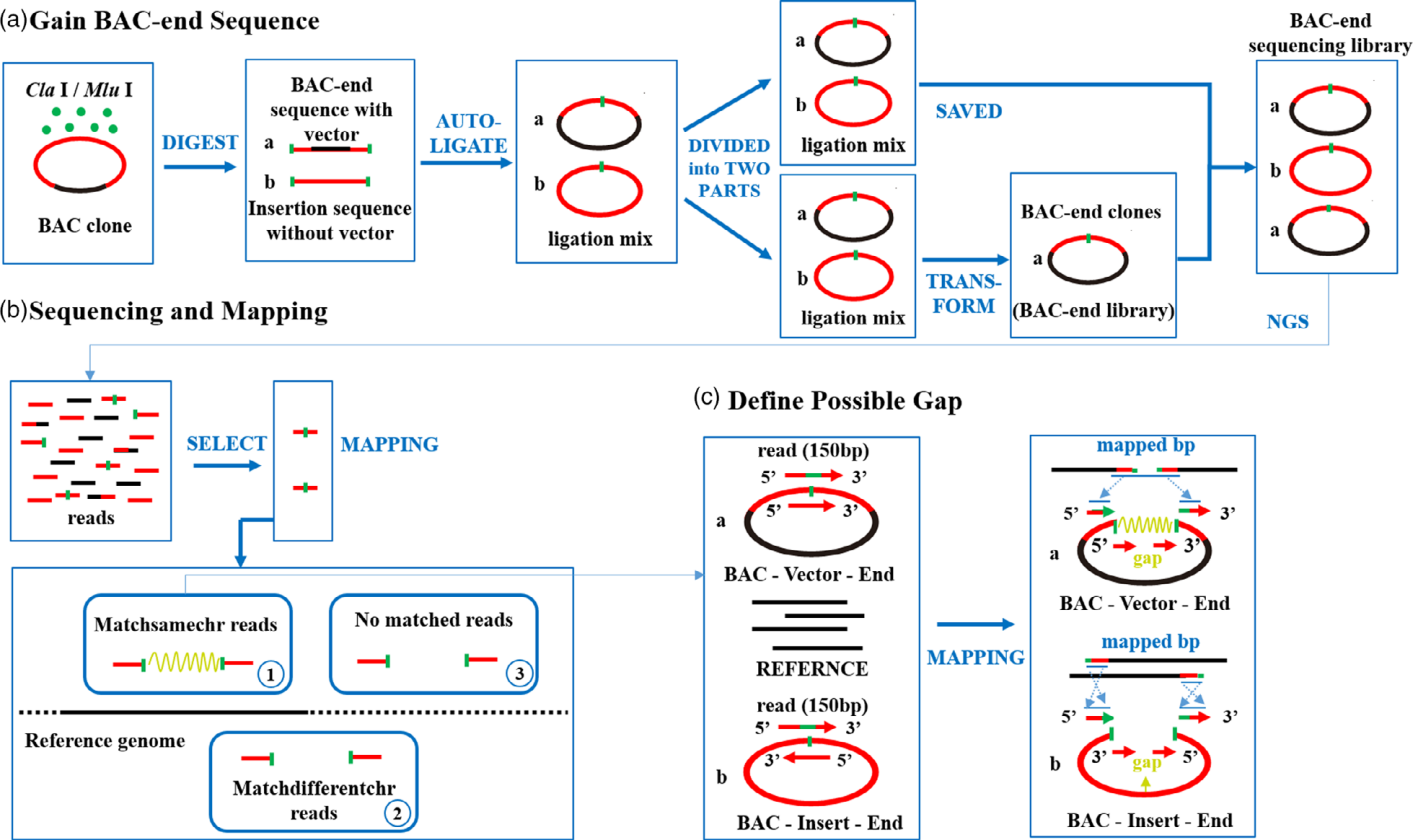
The pipelines of BAC end libraries and BAC‐anchor analysis. (a) The construction of BAC end libraries; (b) sequencing, mapping and identification of three specific types of uniquely mapped reads; (c) define possible gap based on the Matchsamechr reads.

The BAC‐anchor (BAC end analysis protocol) was developed using the Illumina high‐throughput sequencing platform. Rather than directly sequencing all of the BAC end sequences from each BAC clone using Sanger sequencing, our approach focused on the identification of high‐throughput sequencing reads carrying restriction enzyme cutting sites. In brief, we used the gapped mapping protocol to align reads carrying enzyme cutting sites to draft chromosome sequences, scaffolds and/or the genome sequences of a doubled‐haploid line, which enabled us to search for paired‐end reads containing large internal gaps (Figure 1).

Next, three specific types of uniquely mapped reads were identified from BAC end reads or insertion reads as follows (Figure 1b): (i) Matchsamechr reads, in which the sequences at both ends of the restriction enzyme cutting sites were aligned to the same chromosome of the reference genome sequence; (ii) Matchdifferentchr reads, in which the sequences at both ends of the restriction enzyme cutting sites were aligned to different chromosomes of the reference genome sequence, or only one of the two ends was aligned to the reference genome sequence, while the other end was mapped to the gap or unmapped; (iii) No matched reads, in which both ends of restriction enzyme cutting sites were unmapped to the reference genome sequence. Matchsamechr reads provided rich information for the BAC library quality estimation and genomic survey analyses. Matchdifferentchr reads and no matched reads were further used to guide the assembly of unassembled contig/scaffold sequences based on the reads corresponding to genomic sequences on reference chromosome regions; these reads will be significant for further assembly of complex polyploid genomes.

We then applied the BAC end analysis protocol to profile a high‐coverage C88 potato BAC library constructed in our previous study (Yang *et al*., 2015). In total, around 307 200 BAC clones with an average insert size of 90 kb were collected in eight pools. Of these, four DNA pools were extracted and digested completely with the restriction enzyme *Cla* I, and the other four were digested by *Mlu* I. After autoligation and transformation, we obtained eight BAC end libraries containing >3 200 000 transformed BAC end clones (400 000 clones for each pool), including the C88 genome Insertion sequences. The mixed DNA samples were extracted for sequencing using the Illumina HiSeq X10 platform with the 150‐bp paired‐end protocol. A total of 575.66 m clean reads and 172.69 Gb clean data were generated. Using sambamba analysis (Kim *et al*., 2015), we determined a ~59.68× average sequencing depth (~7.46× per sample) in all samples (Table S1).

### BAC end library profiling and insertion length distribution of BAC end libraries

We searched for specific reads with enzyme cutting sites and selected 33 859 069 reads carrying *Cla* I cutting sites and 24 915 443 reads derived from *Mlu* I. After removing the duplicate reads, a total of 9 819 130 reads derived from *Cla* I and 6 228 861 reads derived from *Mlu* I were obtained (Table 1). We then aligned these specific reads to the doubled‐monoploid potato DM1‐3 516 R44 (hereafter referred to as DM) reference genome from ENSEMBL release SolTub3.0 (Bolser *et al*., 2017) using Bowtie 2 (Langmead and Salzberg, 2012) to search for uniquely mapped reads. A total of 1 549 145 BAC end reads and 445 813 insertion reads carrying *Cla* I cutting sites were uniquely mapped to the same chromosome of reference genome. Considering the 24 915 443 reads carrying *Mlu* I, a total of 438 688 BAC end reads and 194 327 insertion reads were uniquely mapped. Hence, the ratios of Matchsamechr unique reads are 20.32% for *Cla* I and 10.16% for *Mlu* I, respectively. The reads which uniquely mapped to different chromosomes (Matchdifferentchr reads) were 2 433 257 and 1 061 139 derived from *Cla* I and *Mlu* I, respectively. Hence, 45.10% of reads derived from *Cla* I and 27.20% derived from *Mlu* I were generated. No matched reads with the DM reference genome sequence numbered 22 046 957 and 18 167 773 derived from *Cla* I and *Mlu* I, respectively (Table 1).

**Table 1:** Statistics of different types of mapped reads

Type of mapped reads		Numbers of reads from *Cla* I	Numbers of reads from *Mlu* I
Total reads carrying the enzyme cutting sites		33 859 069	24 915 443
Unique reads carrying the enzyme cutting sites after removing the duplicates		9 819 130	6 228 861
Matchsamechr unique reads	Total BAC end reads	1 549 145	438 688
	Total insertion reads	445 813	194 327
Matchdifferentchr unique reads		2 433 257	1 061 139
No matched reads		22 046 957	18 167 773
Mapped reads/total unique reads		45.10%	27.20%

**Figure 2:**
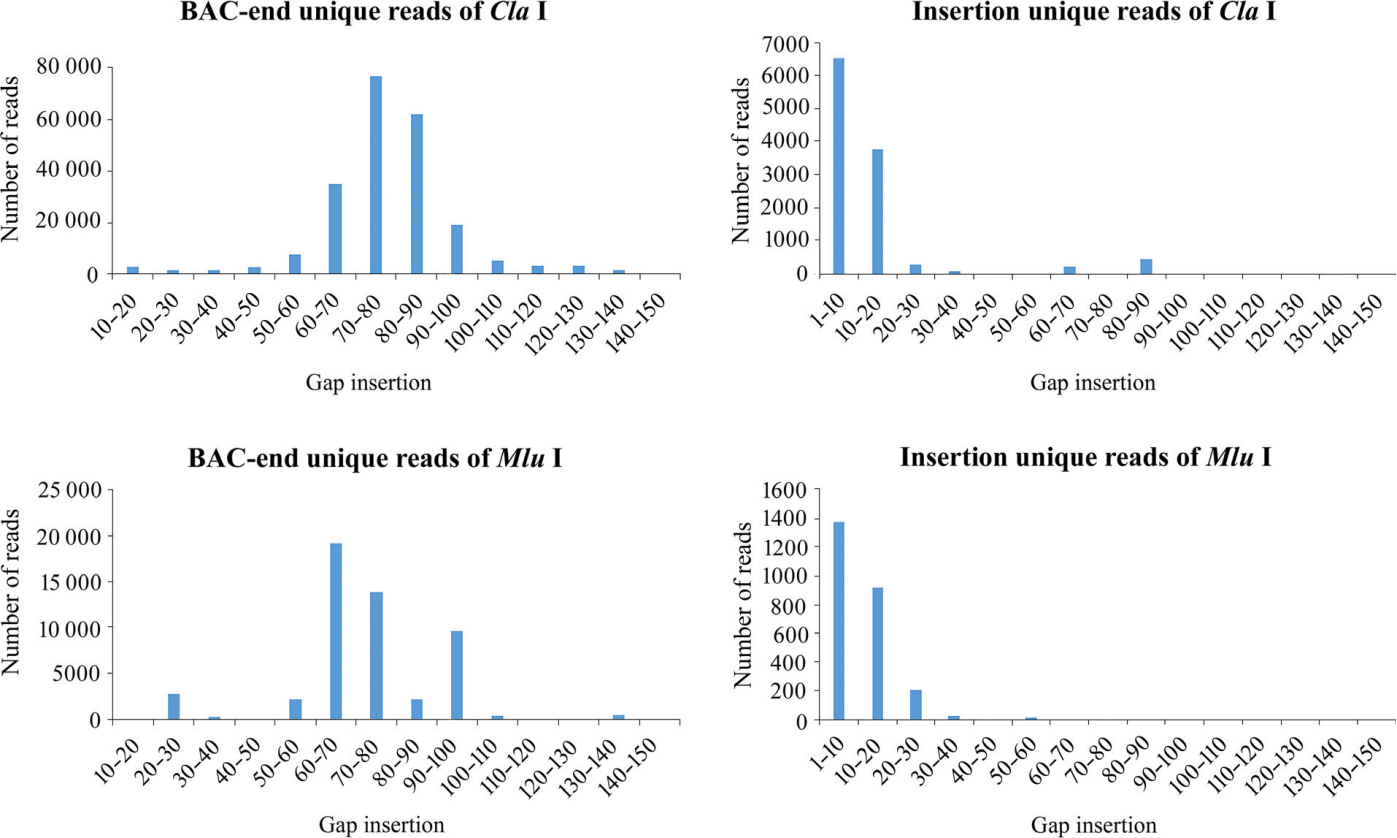
Gap mapped situation of BAC end and genomic reads based on alignment on the potato DM genome sequence.

We then searched for uniquely mapped reads distributed in the 1–150 kb gap insertion. For the 33 859 069 reads carrying *Cla* I cutting sites, a total of 1 353 440 BAC end reads containing a 1–150 kb gap insertion were multi‐mapped on the reference and 852 224 BAC end reads were uniquely mapped. There were 194 361 (22.81%) unique reads generated with an internal gap of 60–100 kb, 77 251 reads containing a 70–80 kb gap, 62 421 reads containing an 80–90 kb gap and 35 264 reads containing a 60–70 kb gap. There were 627 506 unique reads with an insertion of <10 kb and 14 336 reads with an insertion over 100 kb. For insertion reads, 11 506 reads were uniquely mapped to the reference genome with a 1–150 kb gap insertion. In total, 10 298 (89.50%) reads had a 1–20 kb gap distribution on the reference genome sequence (Table 2; Figure 2). The detailed mapping information of unique mapped reads carrying *Cla* I is shown in Table S2.

**Table 2:** Mapped gaps of reads before and after restriction enzyme cutting site alignment to the potato DM reference genome sequence

Internal Gap (kb)	BAC end reads				Insertion reads			
	*Cla* I		*Mlu* I		*Cla* I		*Mlu* I	
	Multi‐mapped	Unique mapped	Multi‐mapped	Unique mapped	Multi‐mapped	Unique mapped	Multi‐mapped	Unique mapped
1–10	981 160	627 506	185 908	131 947	21 361	6519	27 372	1376
10–20	13 504	2848	494	5	9282	3779	7249	923
20–30	11 362	1306	16 448	2732	5403	303	427	206
30–40	6901	1231	416	163	2220	65	813	24
40–50	7680	2957	19 214	44	3267	47	1346	1
50–60	13 152	7679	15 363	2180	2277	26	345	19
60–70	43 461	35 264	20 709	19 262	2072	234	186	6
70–80	94 860	77 251	69 203	13 816	2810	7	402	3
80–90	128 563	62 421	3092	2179	33 891	459	166	3
90–100	28 841	19 425	17 827	9643	1692	9	69	4
100–110	9816	5388	1676	295	3796	6	31	0
110–120	5821	3585	218	24	1442	12	18	0
120–130	5454	3602	132	0	985	1	25	4
130–140	2114	1295	3024	536	1326	5	29	10
140–150	751	466	209	52	765	34	102	0
Total	1 353 440	852 224	353 933	182 878	92 589	11 506	38 580	2579

For the 24 915 443 reads carrying *Mlu* I, a total of 353 933 BAC end reads were multi‐mapped to the reference genome with a 1–150 kb gap insertion and 182 878 BAC end reads were uniquely mapped; 131 947 (72.15%) reads had a 1–10 kb gap distribution, and only 44 900 reads (24.55%) were mapped to the 60–100 gap insertion. For Insertion sequences, a total of 2579 reads had a 1–150 kb gap insertion with 2505 reads of 1–30 kb distribution (Table 2; Figure 2). The detailed mapping information of unique mapped reads carrying *Mlu* I is shown in Table S3.

### Distribution of BAC end reads with a 60–100 kb internal gap on the chromosomes of the potato DM reference genome

The distribution of BAC end reads with a 60–100 kb internal gap on each chromosome was further analysed (Table 3). The number of BAC end reads was highly variable on different chromosomes. A total of 157 410 reads were generated from *Cla* I enzyme digestion with a range of 1275–83 632 reads on chromosomes 00–12. After removing repeat reads at the same sites, a total of 5341 unique reads were mapped to the reference genome with 140–1033 separate unique reads. The total number of BAC end reads with a 60–100 kb internal gap from *Mlu* I was only 20 919, and there are only 165 unique reads. No reads were present on chromosome 4. The highly different distributions of BAC end reads from *Cla* I and *Mlu* I are consistent with the *in silico* enzymatic digestion prediction on the entire genome; the numbers of cutting sites of *Cla* I and *Mlu* I on the entire genome were 138 535 and 15 390, respectively, and the average enzyme segment lengths were 5849 and 52 585 bp, respectively (Figure S1).

**Table 3:** Distribution of BAC end reads containing a 60–100 kb internal gap on the chromosome of the reference genome sequence

Chromo.	Length (bps)	*Cla* I reads		*Mlu* I reads		Non‐duplicate reads with < 100 kb distance between adjacent two BAC ends	Non‐duplicate reads with<100 kb distance between adjacent two BAC ends
		Total	Unique	Total	Unique		
chr00	–	6940	283	5	2	253	30
chr01	88 663 952	6413	471	960	12	343	128
chr02	48 614 681	1921	174	433	4	114	60
chr03	62 190 286	13 981	654	5073	34	563	91
chr04	72 208 621	2315	232	0	0	143	89
chr05	52 070 158	3202	255	939	7	175	80
chr06	59 532 096	5414	367	11 772	19	266	101
chr07	56 760 843	1275	140	2	2	91	49
chr08	56 938 457	3278	166	353	9	110	56
chr09	61 540 751	7194	444	22	15	366	78
chr10	59 756 223	14 728	775	88	24	670	105
chr11	45 475 667	7117	347	126	13	287	60
chr12	61 165 649	83 632	1033	1146	24	937	96
Total	–	157 410	5341	20 919	165	4318	1023

**Table 4:** Aliment category and interlength of Sanger sequencing of BAC‐end clones on the DM reference genome sequence

Alignment category	Interlength (kb)	Numbers of BAC‐end clones
Mapped same chromosome	<1	4
	1–10	4
	30–40	1
	40–50	1
	50–60	2
	60–70	3
	70–80	8
	80–90	10
	90–100	5
	100–110	2
	110–120	2
	120–130	0
	130–140	0
	140–150	0
	>150	9
Mapped different chromosome		46
Only one end aligned		9
No matched		3
Total BAC‐end clones		109

**Figure 3:**
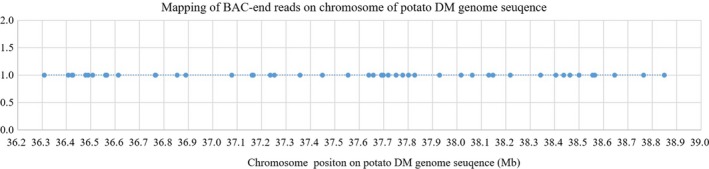
Dense coverage area of BAC end reads with a 60–100 kb internal gap on chromosome 1 of the potato DM genome sequence.

Within the same chromosome, there were enriched and sparse regions covered by the BAC end reads. There were 4318 unique reads with less than 100 kb distance between two adjoined BAC end reads with 91–937 separate reads for 00–12 chromosomes (Table 3). There were 1023 unique reads with more than 100 kb distance between the two adjacent BAC end reads, each with 30 to 128 reads on 12 chromosomes (Table 3). For chromosome 1, a total of 6413 BAC end reads from *Cla* I were mapped on the chromosome with 471 unique reads after removal of repeats. Some mapped regions were denser, while some regions had no reads mapped. In the 36.40–39.00 Mb dense region, multiple BAC end reads per 100 kb were distributed on the chromosome with up to seven BAC end reads (Figure 3), which could build longer contigs or scaffolds based on the average BAC insertion size of 90 kb.

### Validation of HiSeq using Sanger sequencing

In order to further confirm the high‐throughput analysis results, we randomly selected 109 BAC end clones for Sanger sequencing. The paired BAC end (BAC‐PE) sequences from both sides of enzyme cutting sites were then separately aligned to the DM genome assembly (Tables 4, S4) using BlastN. Interlength was used to refer to the distance of mapping locations between two ends of the enzyme cutting sites of Sanger sequences. The results showed that 51 BAC‐PEs aligned to the same chromosomes with the different interlengths of 1–150 kb. Of these, 26 BAC end clones (23.85%) contained 60–100 kb interlengths, which is consistent with the BAC end reads mapping ratio of 22.81% for *Cla* I and 24.55% for *Mlu* I. Moreover, there still two clones (clone68 and clone81) contained the interlengths of 104 960 and 101 014 kb, respectively, which were also consistent with the ~90 kb average insert size of the C88 BAC library. Eight BAC‐PEs, including clone10, clone46 and clone53, were also matched to the same chromosome. However, the interlengths were less than 10 kb, which indicated that there were still gaps between different scaffolds. Fifty‐eight other BAC‐PE sequences were matched on different chromosomes or no match, indicating that there may be big differences between autotetraploid and haploid potato genomes, or that there were errors in the DM genome assembly, which must be further verified.

### SNP and InDel identification

**Figure 4:**
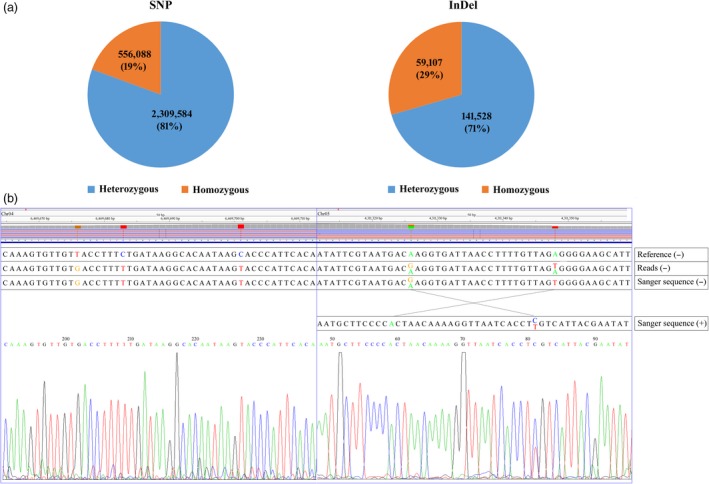
(a) The survey of genomic homogeneity of the autotetraploid potato. (b) The two examples showing the SNPs identified by high‐throughput sequencing and experimental verification by the Sanger sequencing approach. −, antisense chain; +, sense chain.

Next, we surveyed the genomic homogeneity of the autotetraploid potato. We first identified SNPs from the uniquely mapped reads. A total of 2 865 672 SNP sites were identified (Figure 4a), in which 556 088 (19%) sites encoded homogeneous SNPs and 2 309 584 (81%) sites contained heterogeneous SNPs. We then identified a total of 200 635 InDels (Figure 4a). Of these, 141 528 (71%) were heterogeneous InDels. These results showed that C88 is highly heterozygous. These SNPs/InDels could be useful for designing specific primers to screen the BAC library and help to obtain the target BAC clones, which could accelerate full‐length functional gene cloning. Finally, we randomly selected 110 SNPs, including 45 homozygous and 65 heterozygous sites, and verified them by PCR amplification and Sanger sequencing. The alignment with the DM sequence showed that 44 homozygous and 49 heterozygous SNPs (84.55%) were validated (Figure 4b).

## Discussion

Shotgun sequencing strategies such as Illumina NGS, PacBio long‐read sequencing and Hi‐C chromatin interaction mapping are wildly used for whole‐genome *de novo* assembling. Currently, the read length of Illumina Paired‐end platforms is about 300–800 bp. PacBio platforms can generate longer sequencing reads, in which average length often ranged about 15–25 kb. Hi‐C technology provides chromatin contact data and the two‐dimensional genome organization structures of contigs/scaffolds that can be used to cluster contigs/scaffolds into chromosomes. Our BAC‐anchor approach provides 60–100 kb or even 200 kb insertion libraries, which fill a read‐length gap between PacBio platforms and Hi‐C technology. Moreover, BAC‐anchor was sequenced from BAC libraries that provide 60–100 kb or even 200 kb insertion clones for BAC screening, which can cross almost all repeat sequences on the genome and are more effective for the connection of different contigs/scaffolds. This above information could not be obtained from other sequencing approaches. Therefore, BAC‐anchor can be considered as a complementary technology filling the detection gaps between the above platforms that could facilitate the further genome sequencing studies.

Although various paired‐end sequencing methods have been developed to construct long scaffolds from contigs derived from shotgun sequencing, traditional sequencing of BAC libraries still plays an important role in genome assembly. However, sequencing libraries using the Sanger method is expensive and time‐consuming. BAC ends play an important role in the assembly of genomic sequences. Through the double‐terminal sequencing of BAC clones, mate‐pair sequences can be obtained that provide more than 120 kb of ‘rivets’ in the whole‐genome sequence; the scattered contigs can be connected in the draft genome to the longer scaffolds and ultimately to the entire chromosome. Based on digestion and next‐generation sequencing technology, we report here a cost‐effective method for sequencing paired‐end reads of genomic libraries using a tetraploid potato cultivar BAC library as a test case organism.

In this study, we sequenced a total of 307 200 BAC clones. The cost of using the paired‐end Sanger method for this number of clones might have exceeded $3 600 000, while sequencing with our strategy resulted in a 240‐fold reduction in cost (approximately $15 000). Furthermore, the larger the number of BAC clones is, the more the cost ratio is reduced. BAC end Sanger sequencing is also challenging due to the longer insertion fragments (generally more than 100 kb), which result in the formation of more repeats and/or secondary structures that can cause sequencing anomalies. In contrast, our method utilized 150‐bp paired‐end fragments that generally break secondary structures and bioinformatics analyses to filter out repeats and/or multiple mapped sequences. The paired BAC end sequences from these clones will be an important resource for scaffolding forthcoming shotgun sequence assemblies of the crop genomes. Using the Hi‐C software, scaffolds can be assembled with mean lengths of 20 kb, and our BAC‐anchor method can greatly improve scaffolds up to at least 60 kb based on the links of BAC end reads containing a 60–100 kb internal gap. In total, this method provides an important resource for the sequencing and assembly of large and complex genomes.

According to our previous study (Yang *et al*., 2015), the average insert size of the C88 BAC clones was about 90 kb. Among the *Cla* I/*Mlu* I containing BAC end reads that mapped to the same chromosome of potato DM reference genome, only 22.81% of *Cla* I containing reads and 24.55% of *Mlu* I containing reads possessed a 60–100 kb internal gap, while 73.97% *Cla* I and 72.15% *Mlu* containing reads had a 1–20 kb internal gap in this study. The high‐throughput analysis results were verified by sequencing 109 BAC end clones using the Sanger method. The results also showed that about 1/4 of BAC clone sequences were mapped to the reference genome at an average of 90‐kb intervals. Some BAC end sequences that could not be properly aligned to the DM reference genome were also uncovered, suggesting that there were large differences between autotetraploid and haploid genotypes in potato or may exist abundant structural variations in the tetraploid potato genome. We found that 46.79% of BAC end clones were aligned to the same chromosome of reference genome. This ratio is much higher than 20.32% (*Cla* I) and 10.16% (*Mlu* I) values obtained from BAC‐anchor reads. This may be related to the difference of mapping parameters between Bowtie and Blast. Bowtie, a short‐read aligner for short DNA sequences (reads) from next‐generation sequencers, was restricted to align short reads with no more than three mismatched, while the coverage ratio of query length to the total input sequence length would influence the alignment statistics of BAC end Sanger sequences in BlastN. For example, the length of BAC end Sanger sequence longer than 1000 bp (input sequence) and the alignment with query length >300 bp was considered as matched. When we used a higher coverage ratio of >80%, only 23 clones (21.10%) were matched on the same chromosome of reference genome, which is consistent with the BAC‐anchor reads.

Due to the mean insert size of 90 kb in the BAC library, the BAC end reads with an internal gap of over 100 kb indicated some error in the DM genome assembly, but this needs to be further verified. We also identified 2 433 257 and 1 061 139 derived from *Cla* I and *Mlu* I reads, respectively, that mapped to different chromosomes. The no matched reads numbered 22 046 957 and 18 167 773 derived from *Cla* I and *Mlu* I, respectively. These results, especially the Matchdifferentchr reads, will be significant for the assembly of complex polyploid genomes. The occurrence of restriction sites in genes, repetitive elements, or any non‐coding regions can influence the BAC end mapping efficiency. Based on the chromosomes of DM in version 4.04, the size of the potato reference genome was 745 Mb, including coding regions, repeat sequence regions and non‐coding regions of 65.05, 158.38 and 521 Mb, respectively. Taking the BAC end reads with 1–150 bp internal gaps as an example, among the total 852 224 unique reads carrying a *Cla* I site, the number of reads that mapped to the coding regions, repeat sequence regions and non‐coding regions was 208 779, 216 077 and 427 368, respectively. Among the total 182 878 unique reads carrying a *Mlu* I site, the number of reads which mapped to the coding regions, repeat sequence regions and non‐coding regions was 51 626, 26 301 and 104 951, respectively. It can be seen that the reads in the coding regions had a high mapping efficiency. There were 2823 single‐copy loci and 36 205 multiple‐copy loci in the whole DM genome. Among the 852 224 unique reads carrying *Cla* I cutting sites, the reads which mapped to single‐copy genes and multiple‐copy genes were 103 814 and 200 402, respectively, Additionally, for the 182 878 unique reads with *Mlu* I cutting sites, the reads which were located in single‐copy genes and multiple‐copy genes were 20 740 and 44 063, respectively. It can be seen that reads found in single‐copy genes in the reference genome had higher BAC end reads mapping efficiency than genes found in multiple copies.

The selection of restriction enzymes directly affects the distribution of obtained reads. The selected restriction enzyme should have no cutting sites in the BAC vector, shorter target recognition sequences and contain numerous, evenly distributed cutting sites on the reference genome. In this study, we used two enzymes, *Cla* I and *Mlu* I, and we found that the number of reads obtained by these two enzymes was very different. The number of BAC end unique reads with 1‐150 kb internal gaps obtained by *Cla* I was 4.7‐fold higher than those obtained by *Mlu* I. We also performed *in silico* enzymatic analysis using other enzymes, and the results showed that there were significant differences in the cutting sites on the reference genome. Therefore, to better use the BAC‐anchor method, it is important to use electronic enzymatic cutting analysis in order to better select restriction enzymes. Many chromosome regions had no BAC end read coverage in this study. The reason for the lack of reads in these regions may be related to the uneven distribution of *Cla* I/*Mlu* I enzyme sites throughout the genome. This limitation can be improved by increasing the number of restriction enzymes used in the analysis.

Based on next‐generation sequencing and the BAC end strategy developed here, a total of 2 865 672 SNP sites and 200 635 InDels were identified in this study, they were further verified by the tetraploid genome PCR and Sanger sequencing, and the verified result showed high validation rate of homozygous (97.78%) and relatively low validation rate of heterozygous sites (75.38%). The main reason of low validation rate of heterozygous sites may be different alleles of an autosomal locus are sequenced concurrently and displayed as an analogue electropherograms, and Sanger sequencing is unable to detect mosaic alleles below a threshold of 15%–20% and can miss a significant proportion of low‐level mosaic mutations (Jamuar *et al*., 2014). According to our result, we still got validation rate >75% of heterozygous sites, which indicates that PCR product direct sequencing could be used to quickly test the heterozygous sites. These SNP/InDels could be utilized for designing specific primers for the screening of the BAC library and helping to obtain target BAC clones; the ultimate goal of this utility is to accelerate full‐length functional gene cloning.

Based on the dense BAC end reads containing 60–100 kb internal gaps in the reference genome, an *in silico* physical map for the chromosome regions harbouring high‐density BAC end reads could be built. Then, the corresponding BAC clones could be anchored to the specific area to construct the physical map and used to associate this region with the target phenotypic character. This would allow for a target gene to be quickly cloned.

We anticipate our BAC‐anchor protocol will be highly efficient for genome assembly in polyploid crops, especially in allopolyploid crops. The data available in this study, however, are not enough for phasing or haplotyping of autopolyploid crops due to the short reads derived from the second‐generation sequencing platform. To our knowledge, phasing or haplotyping for autopolyploid crops has not been reported yet. Based on our BAC‐anchor method, long BAC end sequencing reads can be obtained using PacBio long‐read sequencing (or Nanopore Technologies) instead of the Illumina PE150 method. The long reads could provide a more effective match to the genome sequence and facilitate phasing or haplotyping in autopolyploid crops.

## Experimental procedures

### Plant material

The plant used in this study was Cooperation‐88 (C88) from southwest China. It is a high‐yielding autotetraploid potato cultivar with durable resistance to *Phytophthora infestans* and excellent virus resistance (Li *et al*., 2011).

### BAC end library construction

The high‐coverage BAC library for the C88 genome was constructed using the CopyControl^TM^ pCC1 BAC‐cloning vector in our previous study (Yang *et al*., 2015). Approximately 307 200 BAC clones were harvested and saved in 800 384‐well plates. All of these BAC clones were grouped into eight pools with each pool containing 38 400 clones, and total DNA was extracted from each pool. Then, the DNAs from four pools were completely digested with the restriction enzyme *Cla* I (New England Biolabs, Ipswich, MA) with the cleavage site ‘5’…AT^CGAT…3’’, and the other four were digested by *Mlu* I (New England Biolabs) with the cleavage site ‘5’…A^CGCGT…3’’. After digestion, two kinds of sequences were generated: BAC end sequences ligated into the pCC1BAC‐cloning vector (hereafter referred to as BAC end sequence) and Insertion sequences without the pCC1BAC‐cloning vector (hereafter referred to as Insertion sequence). The digested sequences were autoligated, and the ligation mixes were obtained. The autoligated directions of BAC end sequences and Insertion sequences were unexpectedly opposite. The ligation mix was divided into two parts, of which one part was saved for direct sequencing. The other part was transferred into EPI300^™^ Electrocompetent *E. coli* (Epicentre), spread out on Luria–Bertani (LB) solid medium containing EPI Induction Solution and 1% (v/v) chloromycetin as an indicator substrate and incubated for 16 h at 37°C. Insertion sequences without vector could not be transformed, so more than 3 200 000 transformed BAC end clones (each BAC end library contained over 400 000 transformed BAC end clones) were obtained and DNAs were extracted. These BAC end DNAs and the first part of the ligation mix DNAs were then mixed together and used for next‐generation sequencing (Figure 1a). Hence, the sequencing library constructed here was actually the mixture of transformed BAC end clones and Insertion sequences.

### Next‐generation sequencing and BAC‐anchor analysis

Sequencing was performed using the Illumina HiSeq X10 platform with the 150‐bp paired‐end protocol. The genome reference sequence and annotation release SolTub3.0 of the potato doubled‐monoploid DM1‐3 516 R44 (hereafter referred to as DM) were downloaded from the ENSEMBL Plants database (ftp://ftp.ensemblgenomes.org/pub/plants/release-34/fasta/solanum_tuberosum/dna/) (Bolser *et al*., 2017).

Our approach focused on the identification of high‐throughput sequencing reads carrying restriction enzyme cutting sites. After adapter filtering and quality assessment, reads carrying *Cla* I or *Mlu* I cutting sites were selected and further aligned to the potato DM genome sequence using Bowtie 2 with > 18 bp sequence on either end of the enzyme cutting sites (Langmead and Salzberg, 2012). The mapping locations of both ends of sequences at the restriction enzyme cutting sites in the genome were separately recorded and compared (Figure 1b). Internal gap was used to refer to the distance of mapping locations between two ends of the enzyme cutting sites of reads. The parameters ‘–min‐intronlen 1000 –max‐intronlen 150 000’ and ‘—ma 2’ were used to search for internal gaps and partially mapped alignments. The reads generated from BAC end sequences and Insertion sequences can be distinguished by the mapping direction on the reference genome sequence, with the same orientation of BAC end reads and reverse orientation of insertion reads (Figure 1c).

We used sambamba_v0.6.4 with default parameters to estimate average sequencing depth (Kim *et al*., 2015). The SAM‐formatted alignment files were sorted using SAMtools (Cambridge, UK, Li *et al*., 2009). Only the uniquely mapped reads aligned to the reference genome with a mapping quality score (‐q) higher than 30 were retained. We subsequently used BAC‐anchor to analyse the SAM‐formatted alignment files.

### SNP and InDel identification

The uniquely mapped reads were grouped using the Picard Tools (version 2.1.1) AddOrReplaceReadGroups package (http://broadinstitute.github.io/picard/). Duplicate reads were removed using the Picard Tools MarkDuplicates package (http://broadinstitute.github.io/picard/). Alignments with N CIGAR reads were split by GATK (version 3.5) SplitNCigarReads package (McKenna *et al*., 2010). Realignments around InDels were performed using the IndelRealigner package after the target regions were identified using the RealignerTargetCreator package (McKenna *et al*., 2010). SNPs were called with the GATK HaplotypeCaller (‐stand_call_conf 20.0 ‐stand_emit_conf 20.0 ‐mbq 20 ‐mmq 20) and filtered with VCFTools.0.1.15 (–min‐alleles 2 –max‐alleles 2 –minQ 20 –minGQ 20 –minDP 5). We used IGV to browse SNPs, InDels and alignment results (Thorvaldsdóttir *et al*., 2013). SNPs were determined to be homozygous to the reference allele if only one base type was detected at a SNP site in all reads. In contrast, SNPs were defined as heterozygous when two or more base types for one SNP position were detected. For validation, we randomly selected SNPs and designed the primers up‐ and downstream of SNPs with an expected PCR product size of approximately 500 bp and performed PCR amplification of the potato C88 genome DNA and Sanger sequencing. The primer sequences were designed to avoid the locations of SNPs.

### BAC end HiSeq sequence verification using Sanger sequencing

The BAC end high‐throughput analysis results were validated by Sanger sequencing of randomly selected BAC end clones. Several BAC end clones were randomly selected and sequenced by Sanger sequencing using the native sequencing primers of the pCC1 cloning vector. The BAC end sequences from both sides of enzyme cutting sites were used to separately blast against the potato DM reference genome from the ENSEMBL plants using BlastN, and the mapped locations were obtained. Interlength was used to refer to the distance of mapping locations between two ends of the enzyme cutting sites of BAC end cloned Sanger sequences.

### Availability of supporting data

High‐throughput sequencing data sets are available at Genome Sequence Archive (GSA) under accession PRJCA000450.

## Conflict of interest

The authors have no conflict of interests.

## Funding

This work has been supported by the National Natural Science Foundation of China (31561143006, 31801421), Shandong Provincial Natural Science Foundation of China (ZR2016CM27), China Agriculture Research System (CARS‐9), Breeding Program of Shandong Province, China (2017LZGC001) and the Taishan Scholars Program of Shandong Province, China (2016‐2020).

## Authors’ contributions

G.L. and X.Y. conceived this project and designed all research with help from S.H. J.L. provided the BAC‐anchor script and analysed the data sets with assistance from J.L. and J.G. X.Y., Y.Y. and X.G. performed the experiments under the supervision of G.L., L.J and D.D. G.L., J.L. and X.Y. wrote the article.

## Supporting information


**Figure S1** Computational evaluation of *in silico* enzymatic cutting on the potato DM genome sequence program.

**Table S1** Basic summary of sequence and sequencing reads mapping to the reference genome.

**Table S2** The detailed mapping information of unique mapped reads carrying *Cla* I cutting sites with the distribution of 1–150 kb gap insertion.

**Table S3** The detailed mapping information of unique mapped reads carrying *Mlu* I cutting sites with the distribution of 1–150 kb gap insertion.

**Table S4** Sanger sequencing of BAC end clones and alignment with the DM reference genome sequence.

## Data Availability

The source code of BACAnchor.0.7 is available at https://pan.baidu.com/s/1CTyEBWjP2T9FIjFq_voa0A.
