# Supplementary figures and images for: A high‐throughput BAC end analysis protocol (BAC‐anchor) for profiling genome assembly and physical mapping
Source: Plant Biotechnol J. 2019 Jul 15;18(2):364–72. doi: 10.1111/pbi.13203 (PMC6953197; doi:10.1111/pbi.13203)

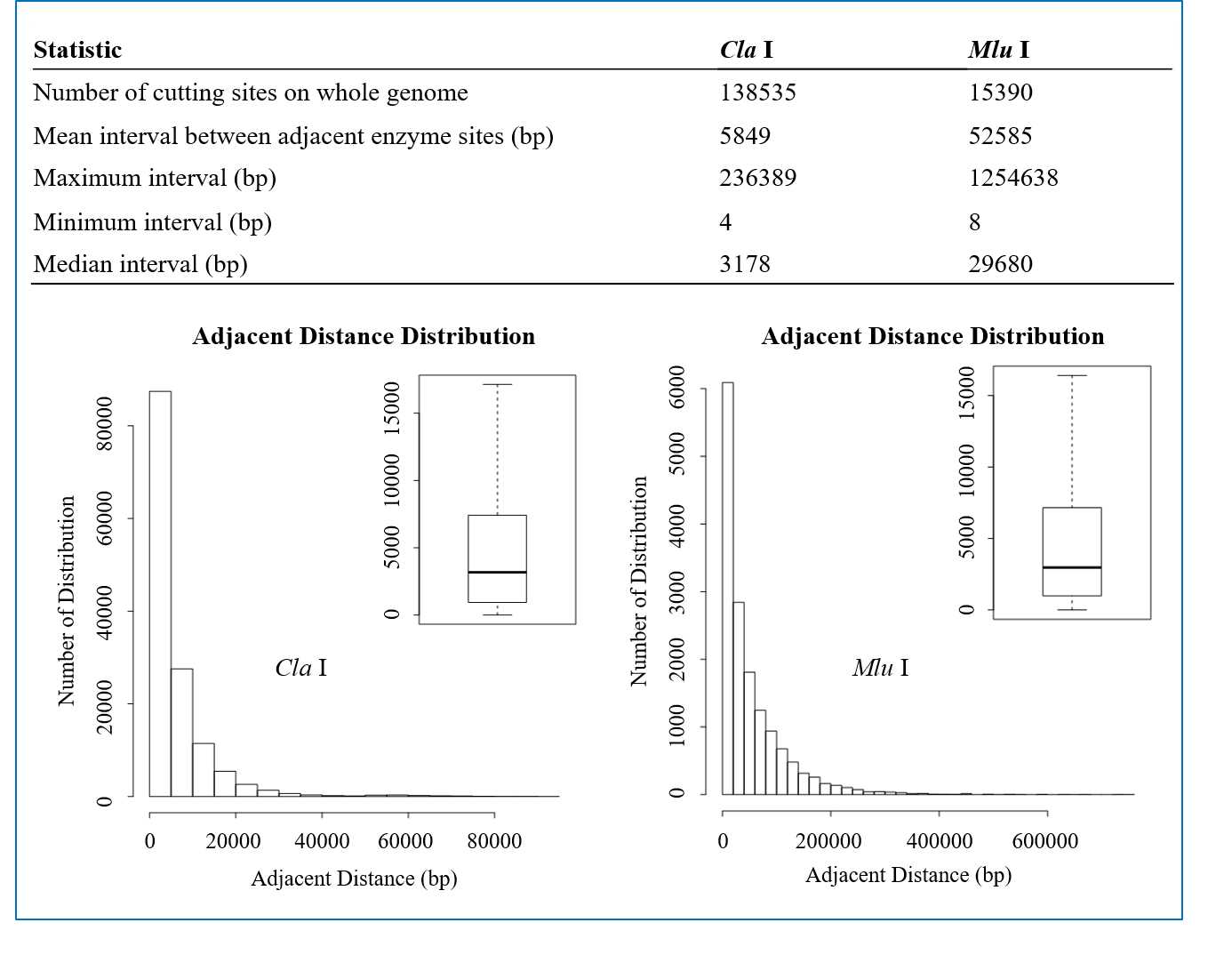

Supplement: Supplementary file 1 — Figure S1 Computational evaluation of in silico enzymatic cutting on the potato DM genome sequence program. [file PBI-18-364-s004.png]
