# Supplementary material for: A high‐throughput BAC end analysis protocol (BAC‐anchor) for profiling genome assembly and physical mapping
Source: Plant Biotechnol J. 2019 Jul 15;18(2):364–72. doi: 10.1111/pbi.13203 (PMC6953197; doi:10.1111/pbi.13203)
Supplement: Supplementary file 2 — Table S1 Basic summary of sequence and sequencing reads mapping to the reference genome. [file PBI-18-364-s005.docx]

**Table S1** Basic summary of sequence and sequencing reads mapping to the reference genome.

| **Library** | **Chromosome** | **Total Reads Bases** | **Aligned Genome Bases** | **Average Depth** |
| --- | --- | --- | --- | --- |
| MluI_library_1 | Chr1 | 334172293 | 43741259 | 7.64 |
| MluI_library_1 | Chr10 | 343233011 | 37613208 | 9.13 |
| MluI_library_1 | Chr11 | 146218360 | 23067828 | 6.34 |
| MluI_library_1 | Chr12 | 425484950 | 38284008 | 11.11 |
| MluI_library_1 | Chr2 | 135836745 | 22005744 | 6.17 |
| MluI_library_1 | Chr3 | 289059382 | 34536750 | 8.37 |
| MluI_library_1 | Chr4 | 184176515 | 31994348 | 5.76 |
| MluI_library_1 | Chr5 | 211560608 | 29244233 | 7.23 |
| MluI_library_1 | Chr6 | 237744144 | 32630051 | 7.29 |
| MluI_library_1 | Chr7 | 137572130 | 24094550 | 5.71 |
| MluI_library_1 | Chr8 | 157656903 | 25112840 | 6.28 |
| MluI_library_1 | Chr9 | 260053482 | 33907445 | 7.67 |
| MluI_library_2 | Chr1 | 446653080 | 46771497 | 9.55 |
| MluI_library_2 | Chr10 | 469737718 | 38689724 | 12.14 |
| MluI_library_2 | Chr11 | 197737719 | 24149291 | 8.19 |
| MluI_library_2 | Chr12 | 587489856 | 40059035 | 14.67 |
| MluI_library_2 | Chr2 | 180166833 | 23478729 | 7.67 |
| MluI_library_2 | Chr3 | 391002615 | 36030907 | 10.85 |
| MluI_library_2 | Chr4 | 237456661 | 34633552 | 6.86 |
| MluI_library_2 | Chr5 | 273914753 | 30283842 | 9.04 |
| MluI_library_2 | Chr6 | 318125226 | 34279015 | 9.28 |
| MluI_library_2 | Chr7 | 176541720 | 25533842 | 6.91 |
| MluI_library_2 | Chr8 | 213606714 | 27473622 | 7.77 |
| MluI_library_2 | Chr9 | 367891501 | 35542818 | 10.35 |
| MluI_library_3 | Chr1 | 315709865 | 41248365 | 7.65 |
| MluI_library_3 | Chr10 | 268337695 | 35779704 | 7.50 |
| MluI_library_3 | Chr11 | 137186346 | 21281722 | 6.45 |
| MluI_library_3 | Chr12 | 439269239 | 36944279 | 11.89 |
| MluI_library_3 | Chr2 | 108147074 | 20776883 | 5.21 |
| MluI_library_3 | Chr3 | 220887491 | 32800751 | 6.73 |
| MluI_library_3 | Chr4 | 162532929 | 30380158 | 5.35 |
| MluI_library_3 | Chr5 | 161715228 | 28029064 | 5.77 |
| MluI_library_3 | Chr6 | 156397443 | 31518264 | 4.96 |
| MluI_library_3 | Chr7 | 86611102 | 22057575 | 3.93 |
| MluI_library_3 | Chr8 | 110005161 | 23567901 | 4.67 |
| MluI_library_3 | Chr9 | 234524018 | 32098475 | 7.31 |
| MluI_library_4 | Chr1 | 284894641 | 36061302 | 7.90 |
| MluI_library_4 | Chr10 | 233508477 | 33467616 | 6.98 |
| MluI_library_4 | Chr11 | 121065595 | 18526870 | 6.53 |
| MluI_library_4 | Chr12 | 393065012 | 34463886 | 11.41 |
| MluI_library_4 | Chr2 | 93290076 | 18317616 | 5.09 |
| MluI_library_4 | Chr3 | 193021439 | 30185409 | 6.39 |
| MluI_library_4 | Chr4 | 140503614 | 26734414 | 5.26 |
| MluI_library_4 | Chr5 | 132127977 | 25027483 | 5.28 |
| MluI_library_4 | Chr6 | 122916514 | 28307198 | 4.34 |
| MluI_library_4 | Chr7 | 72725286 | 19591419 | 3.71 |
| MluI_library_4 | Chr8 | 91976019 | 20555919 | 4.47 |
| MluI_library_4 | Chr9 | 200884971 | 29116281 | 6.90 |
| ClaI_library_1 | Chr1 | 324135353 | 41075443 | 7.89 |
| ClaI_library_1 | Chr10 | 366494464 | 34387981 | 10.66 |
| ClaI_library_1 | Chr11 | 151966936 | 21230285 | 7.16 |
| ClaI_library_1 | Chr12 | 417457042 | 35052133 | 11.91 |
| ClaI_library_1 | Chr2 | 154924215 | 21876757 | 7.08 |
| ClaI_library_1 | Chr3 | 299046474 | 32066775 | 9.33 |
| ClaI_library_1 | Chr4 | 180957797 | 29344348 | 6.17 |
| ClaI_library_1 | Chr5 | 221001447 | 25965696 | 8.51 |
| ClaI_library_1 | Chr6 | 243182766 | 30087619 | 8.08 |
| ClaI_library_1 | Chr7 | 139940338 | 22119117 | 6.33 |
| ClaI_library_1 | Chr8 | 159006571 | 22721896 | 7.00 |
| ClaI_library_1 | Chr9 | 258339855 | 31272767 | 8.26 |
| ClaI_library_2 | Chr1 | 271609900 | 37488614 | 7.25 |
| ClaI_library_2 | Chr10 | 280439158 | 31975521 | 8.77 |
| ClaI_library_2 | Chr11 | 130945743 | 18938407 | 6.91 |
| ClaI_library_2 | Chr12 | 325003049 | 33083804 | 9.82 |
| ClaI_library_2 | Chr2 | 107735934 | 18575046 | 5.80 |
| ClaI_library_2 | Chr3 | 262080288 | 29858118 | 8.78 |
| ClaI_library_2 | Chr4 | 152992655 | 26781529 | 5.71 |
| ClaI_library_2 | Chr5 | 175326827 | 24341399 | 7.20 |
| ClaI_library_2 | Chr6 | 214648288 | 27876385 | 7.70 |
| ClaI_library_2 | Chr7 | 119446337 | 20164259 | 5.92 |
| ClaI_library_2 | Chr8 | 126102088 | 21472498 | 5.87 |
| ClaI_library_2 | Chr9 | 213583540 | 28594228 | 7.47 |
| ClaI_library_3 | Chr1 | 284199799 | 35205476 | 8.07 |
| ClaI_library_3 | Chr10 | 314398108 | 31192768 | 10.08 |
| ClaI_library_3 | Chr11 | 129046772 | 18198288 | 7.09 |
| ClaI_library_3 | Chr12 | 360102734 | 32246981 | 11.17 |
| ClaI_library_3 | Chr2 | 124324561 | 17986858 | 6.91 |
| ClaI_library_3 | Chr3 | 258264161 | 29031417 | 8.90 |
| ClaI_library_3 | Chr4 | 159759617 | 24991726 | 6.39 |
| ClaI_library_3 | Chr5 | 193641737 | 23411943 | 8.27 |
| ClaI_library_3 | Chr6 | 231770736 | 26977132 | 8.59 |
| ClaI_library_3 | Chr7 | 133224688 | 18799825 | 7.09 |
| ClaI_library_3 | Chr8 | 152632491 | 19955369 | 7.65 |
| ClaI_library_3 | Chr9 | 240434355 | 27920844 | 8.61 |
| ClaI_library_4 | Chr1 | 210110796 | 31466038 | 6.68 |
| ClaI_library_4 | Chr10 | 216971780 | 28739654 | 7.55 |
| ClaI_library_4 | Chr11 | 95794345 | 16652519 | 5.75 |
| ClaI_library_4 | Chr12 | 256979386 | 30041524 | 8.55 |
| ClaI_library_4 | Chr2 | 84278011 | 15702288 | 5.37 |
| ClaI_library_4 | Chr3 | 198759723 | 25935507 | 7.66 |
| ClaI_library_4 | Chr4 | 121298387 | 22215604 | 5.46 |
| ClaI_library_4 | Chr5 | 134535081 | 21362838 | 6.30 |
| ClaI_library_4 | Chr6 | 177513677 | 24824896 | 7.15 |
| ClaI_library_4 | Chr7 | 87160248 | 16756043 | 5.20 |
| ClaI_library_4 | Chr8 | 97258624 | 18287043 | 5.32 |
| ClaI_library_4 | Chr9 | 175986486 | 24962005 | 7.05 |
| Average | - | 218116765.6 | 28116790.66 | 7.46* |
| *The alignments with "Cigar N" were remove from the analysis. The average coverage of the 8 libraries is about 7.46. | | | | |
