# Supplementary material for: A high‐throughput BAC end analysis protocol (BAC‐anchor) for profiling genome assembly and physical mapping
Source: Plant Biotechnol J. 2019 Jul 15;18(2):364–72. doi: 10.1111/pbi.13203 (PMC6953197; doi:10.1111/pbi.13203)
Supplement: Supplementary file 5 — Table S4 Sanger sequencing of BAC end clones and alignment with the DM reference genome sequence. [file PBI-18-364-s001.docx]

**Table S4** Sanger sequencing of BAC-end clones and alignment with the DM reference genome sequence.

| BAC end clone | Alignment with reference sequence | | | |
| --- | --- | --- | --- | --- |
|  | 5' enzyme cutting site | 3' enzyme cutting site | Alignment category | Insertion length (bp) |
| clone1 | 10:37854853-37855748 | 1:49841624-49842475 | different chr |  |
| clone2 | 9:42569524-42570267 | 12:8427520-8428368 | different chr |  |
| clone3 | 10:37854853-37855735 | 1:49841527-49842475 | different chr |  |
| clone4 | 8:6261315-6262239 | 1:26065225-26065657 | different chr |  |
| clone5 | 9:635537-636143 | 9:579809-580349 | same chr | 56,334 |
| clone6 | 8:6261432-6262239 | 9:10693337-10694194 | different chr |  |
| clone7 | 1:49841732-49842475 | 12:8427520-8428378 | different chr |  |
| clone8 | 7:5384040-5384739 | 10:31430831-31431563 | different chr |  |
| clone9 | 10:31430818-31431563 | 1:47993333-47993887 | different chr |  |
| clone10 | 5:36904493-36905288 | 5:36897631-36898038 | same chr | 7,657 |
| clone11 | 12:8346671-8347580 | 12:8426631-8427525 | same chr | 79,051 |
| clone12 | 3:15372354-15373264 | 3:15454558-15455004 | same chr | 82,650 |
| clone13 | 12:8427520-8428456 | 9:42569524-42570267 | different chr |  |
| clone14 | 3:28067632-28068561 | 3:28140857-28141700 | same chr | 73,225 |
| clone15 | 10:7127068-7127795 | 10:21997918-21998578 | same chr | 49,279,877 |
| clone16 | 3:30245078-30245962 | 4:34486659-34487467 | different chr |  |
| clone17 | 1:19257111-19257860 | 1:19321667-19322222 | same chr | 65,111 |
| clone18 | 4:13093749-13094229 | 4:13174401-13174895 | same chr | 81,146 |
| clone19 | 1:12425800-12426220 | -- | only one end aligned |  |
| clone20 | 10:24301133-24301735 | 10:24392508-24392784 | same chr | 91,651 |
| clone21 | -- | 4:41099863-41099957 | only one end aligned |  |
| clone22 | 2:2550361-2550710 | 2:891022-891363 | same chr | 1,659,339 |
| clone23 | 7:39264822-39265386 | 12:2525139-2525595 | different chr |  |
| clone24 | 8:12305101-12305446 | 5:34487479-34487777 | different chr |  |
| clone25 | -- | -- | no match |  |
| clone26 | 1:19307068-19307117 | 1:19257101-19257860 | same chr | 50,016 |
| clone27 | 3:12638722-12639063 | 3:12432328-12432625 | same chr | 206,735 |
| clone28 | 3:20136445-20136682 | 5:33354399-33354499 | different chr |  |
| clone29 | 8:23536468-23536941 | 8:23616044-23616847 | same chr | 80,379 |
| clone30 | 4:47781678-47781902 | 4:47860615-47860969 | same chr | 79,291 |
| clone31 | 8:37263142-37263586 | 4:70277721-70277989 | different chr |  |
| clone32 | 11:33887919-33888365 | 11:42134202-42135048 | same chr | 8,246,283 |
| clone33 | 9:6216192-6216696 | 3:15925591-15925874 | different chr |  |
| clone34 | 1:35090325-35090960 | 1:35024693-35025594 | same chr | 66,267 |
| clone35 | 8:44395496-44395917 | 8:48483456-48483708 | same chr | 4,088,212 |
| clone36 | 10:20872947-20873032 | 10:20971922-20972013 | same chr | 99,066 |
| clone37 | 6:35607784-35608228 | 8:7651479-7651758 | different chr |  |
| clone38 | 12:12241120-12241500 | 12:12164709-12164940 | same chr | 76,791 |
| clone39 | 4:41099863-41099957 | -- | only one end aligned |  |
| clone40 | 11:13390524-13391129 | 11:13468356-13469057 | same chr | 78,533 |
| clone41 | 2:22881140-22881619 | 12:43957986-43958150 | different chr |  |
| clone42 | 1:26065225-26065657 | 11:10983657-10984255 | different chr |  |
| clone43 | -- | 1:19257101-19257820 | only one end aligned |  |
| clone44 | 2:9084184-9084822 | 2:9009872-9010152 | same chr | 74,950 |
| clone45 | 8:19000573-19000816 | 8:19076069-19076843 | same chr | 76,270 |
| clone46 | 1:35827845-35828032 | 1:35827593-35827850 | same chr | 439 |
| clone47 | 4:34486777-34487583 | 1:47982552-47983208 | different chr |  |
| clone48 | 8:26239024-26239289 | 3:33181705-33181867 | different chr |  |
| clone49 | 8:37263105-37263740 | 7:10982366-10982755 | different chr |  |
| clone50 | 1:44848803-44848904 | 10:822353-822634 | different chr |  |
| clone51 | 2:29252552-29253105 | 4:41739267-41739797 | different chr |  |
| clone52 | 4:36966943-36967453 | 2:9705551-9706104 | different chr |  |
| clone53 | 12:44614395-44614813 | 12:44614395-44614813 | same chr | 973 |
| clone54 | -- | -- | no match |  |
| clone55 | 10:57016615-57017491 | 10:57018889-57019809 | same chr | 3,194 |
| clone56 | 2:653407-653905 | 8:15421750-15421956 | different chr |  |
| clone57 | 2:20462408-20463083 | 2:20365012-20365077 | same chr | 98,071 |
| clone58 | 5:21021938-21022492 | 2:17123296-17123390 | different chr |  |
| clone59 | 1:35827845-35828032 | 1:35827593-35827850 | same chr | 439 |
| clone60 | 5:32381402-32381464 | 12:17332338-17332784 | different chr |  |
| clone61 | 9:18378274-18378738 | 9:17946766-17947215 | same chr | 431,972 |
| clone62 | 6:45780174-45780301 | -- | only one end aligned |  |
| clone63 | 10:26426241-26426580 | 10:26313437-26314341 | same chr | 113,143 |
| clone64 | 1:50984295-50985124 | 5:26196160-26196600 | different chr |  |
| clone65 | 5:36931384-36932264 | 5:37027073-37027459 | same chr | 96,075 |
| clone66 | 8:50108569-50108697 | 8:50223378-50223809 | same chr | 115,240 |
| clone67 | 8:40364172-40364541 | 1:18892069-18892935 | different chr |  |
| clone68 | 1:16263070-16263108 | 1:16367901-16368030 | same chr | 104,960 |
| clone69 | 7:18126208-18126236 | 11:36414696-36414959 | different chr |  |
| clone70 | 12:25603041-25603483 | 2:7433850-7434414 | different chr |  |
| clone71 | 10:21509789-21510663 | 9:19157975-19158205 | different chr |  |
| clone72 | 10:49360748-49360809 | 1:24653729-24654059 | different chr |  |
| clone73 | 12:45526161-45526929 | 12:45602343-45603009 | same chr | 76,848 |
| clone74 | -- | 5:26196160-26196496 | only one end aligned |  |
| clone75 | 11:5003595-5004141 | 11:5085784-5086433 | same chr | 82,838 |
| clone76 | 6:6446966-6447757 | 6:6487722-6488635 | same chr | 41,669 |
| clone77 | 6:6446985-6447757 | -- | only one end aligned |  |
| clone78 | 12:56417124-56417464 | 12:56483339-56483526 | same chr | 66,402 |
| clone79 | 3:21433277-21434038 | 3:30113236-30113689 | same chr | 8,680,412 |
| clone80 | 5:1058804-1058873 | 1:23690449-23691369 | different chr |  |
| clone81 | 4:49116434-49117040 | 4:49217131-49217448 | same chr | 101,014 |
| clone82 | 7:35798303-35798741 | 7:35868429-35868763 | same chr | 70,460 |
| clone83 | 2:18770730-18770955 | 4:48896385-48896458 | different chr |  |
| clone84 | 12:11735115-11735305 | 5:36152685-36153525 | different chr |  |
| clone85 | 5:21021938-21022492 | 2:17123296-17123390 | different chr |  |
| clone86 | 2:653407-653905 | 8:15421750-15421956 | different chr |  |
| clone87 | 11:32713377-32713720 | 11:32624982-32625138 | same chr | 88,738 |
| clone88 | 9:18378274-18378738 | 9:17946766-17947215 | same chr | 431,972 |
| clone89 | 5:18427720-18428685 | 5:18508715-18508917 | same chr | 81,197 |
| clone90 | 1:14473670-14473999 | 8:50108569-50108697 | different chr |  |
| clone91 | 2:2125509-2126223 | 2:2118009-2118488 | same chr | 8,214 |
| clone92 | 3:12507397-12507589 | 3:12468664-12469125 | same chr | 38,925 |
| clone93 | 5:36904476-36905288 | 5:36897631-36898038 | same chr | 7,657 |
| clone94 | 9:567125-567565 | 10:42869308-42869552 | different chr |  |
| clone95 | 12:32783723-32784290 | 11:10983722-10984255 | different chr |  |
| clone96 | 4:6389526-6390656 | 4:6469629-6470321 | same chr | 80,795 |
| clone97 | 6:14735327-14736051 | 10:23230935-23231424 | different chr |  |
| clone98 | 1:26065971-26066483 | 11:33303813-33304441 | different chr |  |
| clone99 | -- | -- | no match |  |
| clone100 | 4:17102114-17102215 | 1:22710820-22711008 | different chr |  |
| clone101 | 5:17233626-17233778 | 6:49587665-49588093 | different chr |  |
| clone102 | -- | 1:56301757-56301850 | only one end aligned |  |
| clone103 | 1:43435265-43435443 | 8:20893589-20893950 | different chr |  |
| clone104 | 12:8427520-8428148 | 12:8427237-8427525 | same chr | 911 |
| clone105 | 2:36397264-36397516 | 2:36311156-36311608 | same chr | 86,360 |
| clone106 | 12:18609954-18610618 | 12:16386478-16386896 | same chr | 2,224,140 |
| clone107 | 1:35827845-35828032 | -- | only one end aligned |  |
| clone108 | 7:55069184-55069350 | 7:54984238-54984865 | same chr | 85,112 |
| clone109 | 8:21691060-21691671 | 8:21597615-21597691 | same chr | 94,056 |
